# Health insurance policy enforcement and catastrophic health expenditure: a case study in Sichuan province, China

**DOI:** 10.3389/fpubh.2025.1596377

**Published:** 2025-06-03

**Authors:** Wei Liu, Guowu Huang

**Affiliations:** School of Public Administration, Sichuan University, Chengdu, China

**Keywords:** urban and rural residents’ health insurance, compulsory enrollment, incentivized enrollment, deviation in policy enforcement, catastrophic health expenditure, preference for healthcare institution

## Abstract

**Introduction:**

China’s health insurance reforms aim for universal coverage and financial relief, but implementation varies by region, urban–rural areas, and ethnic groups, highlighting disparities in healthcare access and socioeconomic status. Yet, the specific impact of policy enforcement deviations on catastrophic health expenditure remains underexplored, particularly amid China’s urban–rural and ethnic diversity.

**Methods:**

Our study is based on survey data of urban and rural residents’ medical insurance in Chengdu, Zigong, Nanchong, Aba Tibetan, Qiang Autonomous Prefecture, and Liangshan Yi Autonomous Prefecture of Sichuan Province (*N* = 1,460), exploring the impact of deviations in health insurance policy enforcement (DPE) on catastrophic health expenditure (CHE).

**Results:**

By constructing a binary probit model, instrumental variable method, and heterogeneity test, the study finds that DPE significantly increases the risk of households experiencing CHE, with each unit increase in the degree of deviation raising the probability of CHE by 7.45%. These risks are particularly pronounced in rural areas (*p* < 0.05), ethnic minority settlements (*p* < 0.01, *p* < 0.05), and the insured population (*p* < 0.05), with clear superimposed effects of economic vulnerability and cultural differences. Further analysis indicates that residents’ preferences for healthcare institutions (PHI) mediate between DPE and CHE, with policy execution deviations indirectly increasing the medical burden by inducing residents to choose higher-level healthcare institutions.

**Discussion:**

Drawing from empirical findings, we suggest enhancements in three areas: standardizing grassroots policy implementation, refining financing mechanisms, and advancing payment reforms to effectively mitigate CHE risks. Our study offers empirical support for improving the execution of medical insurance policies and driving reforms in healthcare systems, particularly by providing policy insights related to urban–rural integration and rural revitalization.

## 1 Introduction

The continuous deepening of reforms in China’s healthcare security system has led to implementation of basic health insurance systems for urban and rural residents (hereinafter referred to as resident health insurance) as a policy focus. Data from the National Healthcare Security Administration indicates that by the end of 2023, China’s resident health insurance enrollment rate will have stabilized at over 95%, with a year-on-year increase of 21.1% in the number of benefit claims. However, while health insurance coverage has significantly improved, catastrophic health expenditure (CHE) remains serious. Studies have shown that China’s CHE rate has increased (1). According to the 2023 “China Health Statistics Yearbook,” the average proportion of healthcare expenditure to consumer expenditure for urban and rural residents rose to 8.3 and 9.9%, respectively, in 2021, with this issue being particularly pronounced in the western regions. Taking Sichuan Province as an example, the proportions of healthcare expenditure to consumer expenditure for urban and rural residents were 8.5 and 11.4%, respectively. A higher proportion of medical expenses may reduce consumption among other households facing significant medical and financial pressure, thereby triggering the risk of CHE. This contradiction suggests that there may be considerable deviations between the design of a medical insurance policy and its execution, and the risk of deviation in policy enforcement (DPE) requires urgent attention. Public policy execution deviation, which refers to grassroots divergence from the original intentions of the system during policy implementation, is a critical issue in public management. Previous studies have explored public policy enforcement bias from contextual dimensions such as street-level bureaucrats, professional law enforcers, arable land protection, environmental constraints, and government purchases of public services (2–8). In health insurance, DPE primarily appears as a non-standard methods such as mandatory enrollment and financial incentives. In certain regions, village committees implement compulsory apportionment or bundle benefits to achieve enrollment targets, compelling low-income families to pay fees, which diminishes their basic living expenses. In other areas, cash incentives encourage enrollment, leading to “adverse selection”—where healthy individuals join to receive subsidies, while those truly in need struggle to benefit due to information asymmetry or lack of resources competition^1^. Such practices may temporarily increase the enrollment rate but violate the principle of “voluntary enrollment” in resident medical insurance, potentially exacerbating the burden on residents, weakening policy trust, triggering resistance to the medical insurance system among residents, reducing the effectiveness of the policy, and ultimately exacerbating the catastrophic risk of health expenditure.

Existing research has primarily focused on the direct impact of the medical insurance system on CHE, such as updates to system support programs, expense reimbursement, and equity (9–11). There is a clear scarcity of predictors at the community and policy levels (12), with insufficient attention given to the micro-mechanisms by which policy execution losses may affect CHE. Studies have suggested that inadequate grassroots governance capacity, misalignment of power and empowerment, policy information transmission, and satisfaction deviations from government credibility may lead to execution deviations (13–16). There is a lack of empirical evidence to support these claims. Particularly in the western regions, where urban–rural differences are pronounced and ethnic minorities are concentrated, policy execution encounters more complex cultural, economic, and social contexts, and the association between DPE risk and CHE urgently needs clarification. Sichuan Province, a populous western province with characteristic features of both developed cities and underdeveloped ethnic areas, holds significant reference value for the entire country regarding the effectiveness of its medical insurance policy. The “Fourteenth Five-Year Plan for the Development of Medical Security in Sichuan Province” released in 2021, explicitly states the goal of “preventing poverty and re-poverty due to illness.” However, whether grassroots execution deviations become a hidden obstacle to achieving this goal during the actual implementation of a medical insurance policy requires in-depth exploration.

Our study relies on survey data regarding resident health insurance in Chengdu, Zigong, Nanchong, Aba Tibet, the Qiang Autonomous Prefecture, and the Liangshan Yi Autonomous Prefecture in Sichuan Province. We systematically analyzed the impact of DPE on CHE. By constructing a binary probit model, employing the instrumental variable method, and conducting heterogeneity tests, we reveal how mandatory insurance participation and paid incentives increase the risk of CHE through channels that exacerbate the economic burden on residents and undermine policy effectiveness trust.

The contributions of our study are as follows. First, we are the first to incorporate DPE into the CHE analytical framework, expanding the health economics research perspective. Second, by focusing on urban–rural differences in the multi-ethnic regions of the West, we reveal the disruptive factors of DPE on regional development imbalance. Third, we suggest that preferences for healthcare institutions (PHI) have a mediating effect; accordingly, we offer policy insights into promoting tiered medical services. In advancing the “Healthy China 2030” initiative and rural revitalization strategy, we believe it is necessary to strengthen the standardization and transparency of grassroots policy implementation to avoid secondary harm to vulnerable groups due to biased operations, thereby achieving inclusiveness and sustainability of healthcare security.

## 2 Research theory and hypothesis

At the grassroots level, the noncooperation of policy recipients with norms and the complexity of the causal relationship between policy and its impact makes it difficult to adjust public policies. Due to conflicts of interest in the policy implementation process, compliance and performance measurements focus on the contributions of policy recipients and policymakers, respectively, and these two aspects also constitute challenges in policy implementation (17, 18). From the perspective of the relationship between superiors and subordinates, the compliance patterns of subordinates with superiors’ policies are influenced by recognition and administrative pressure. Considering their career development, subordinates may ignore the intentions of central policies and adopt extreme measures to achieve governance goals, even at a high cost, leading to a change like policy implementation (19). Consequently, it is easy to derive manifestations of policy implementation, such as perfunctory behavior, addition, substitution, delay, and brutality (20). Furthermore, the generalization of law enforcement is closely linked to direct pressure at the grassroots level and factors like organizational convergence and unequal distribution of policy implementation capacity (21). Furthermore, the inapplicability of policy reforms can result in the decline of grassroots governance. The comprehension and preferences of policy implementers at the grassroots level also influence governance outcomes (22). These unconventional methods can collectively be referred to as deviations in policy enforcement. Due to the behavioral constraints of policy response, grassroots governments actually play the triple roles of “state agent,” “rational person” and “social agent,” and this has become the inducement to restrain their subjective will to implement policies (23).

As an important component of China’s social security system, resident health insurance provides medical coverage for insured residents. Analyzing from the perspective of the institutional logic of China’s health insurance participation policy, the gap between the top-level design of the policy, the participation policy for special populations, and the idealized policy objectively creates a bias in the actors’ understanding of the policy objectives (24). This in turn, has led to an information disconnect between health insurance policy understanding at the grassroots level between implementers and residents, which is more likely to induce the problem of health insurance de-insurance (25).

In the Chinese context, task indicators for increasing the renewal rate of residents’ health insurance and urging universal health insurance coverage are often linked to the performance appraisal of grassroots executors and their career advancement. Due to the layered pressure to execute and the reality that village committee or community grassroots workers often hold multiple roles, the mismatch between policy tasks and the human resources for execution may cause policy executors to adopt methods such as coercively enrolling residents or offering financial incentives for enrollment to meet task targets. This practice contravenes the policy principle of “voluntary enrollment” in resident health insurance. Analyzing the inherent logic, this approach may exacerbate inequality within the population, leading some residents who initially intended to participate to distrust the village community and resisting enrollment. Residents’ negative attitudes towards the village community can easily generalize to the healthcare policy itself, resulting in alienating attitudes and behaviors towards healthcare policy, and consequently resistance to health insurance enrollment (26), making them more vulnerable to the risk of CHE due to high health costs. Additionally, the aforementioned non-compliant execution methods of the policy may trigger a “taking advantage” mentality among residents, leading some who do not genuinely need expensive medical services to seek more costly services when ill (27) to compensate for the cost void created by insurance premiums. Meanwhile, other residents needing services may not receive adequate protection because of limited resources. Furthermore, without following conventional enrollment methods, residents’ understanding of health insurance may not be clear, and blind or forced enrollment is more likely to place the insured in situations of information asymmetry (28). The violation of policy principles and misaligned policy incentives lead to increased out-of-pocket expenses for the insured (29, 30), leading to the risk of CHE. Therefore, the following preliminary hypothesis is proposed:

*H1a*: Village community enforces mandatory health insurance enrollment can exacerbate residents’ CHE risk.

*H1b*: The “incentive insurance” treatment implemented by the village community will exacerbate residents’ CHE risk.

*H1c*: The higher the level of DPE in the village community, the higher the risk of CHE in individual households.

In general, residents in rural areas face a higher risk of CHE (31), a phenomenon attributable to the interplay of multiple structural factors. The chronic shortage of medical resources in rural areas exacerbates the barriers to accessing healthcare services. Rural households are more prone to CHE in scenarios such as inpatient services, chronic disease management, and older adult care, with incidence rates significantly higher than those in urban households (32). The root of this disparity lies in the generally lower income levels of rural households coupled with rising demand for healthcare due to population aging and a high incidence of chronic diseases, resulting in a higher proportion of medical expenditure relative to household disposable income. Despite increased coverage of residents’ medical insurance, the fragmentation of compensation mechanisms and the inadequate quality of primary healthcare services still expose rural households to high financial risk. The inhibitory effect of medical insurance on CHE exhibits significant heterogeneity and is influenced by the type of insurance, system design, and regional economic conditions. Relevant research indicates that community-based insurance schemes can reduce the risk of CHE by 23.2%, highlighting the financial protection function of these scheme insurance (33). However, cases from South Korea and China reveal a paradox: households enrolled in the national basic medical insurance face a higher CHE (34, 35). This paradox can be explained in two ways: firstly, it is true that health insurance as a compensation mechanism can reduce the burden of disease on patients, but the improvement of health insurance benefits can be due to the existence of moral hazard (36, 37), thus increasing out-of-pocket expenses; secondly, previous study has shown that different types of health care utilization can have a decisive impact on final health care expenditures, for example, individuals who use inpatient and ambulatory services show significant heterogeneity in final health care expenditures (38). If the copayment ratio and cap line design of medical insurance fail to align with household payment capacity, combined with frequent use of medical products, outpatient and inpatient services (39), they may not effectively prevent catastrophic expenditures, creating a gap between the “nominal coverage” and the “actual protection effect” of medical insurance. In other words, under the logic of mandatory enrollment as well as induced enrollment policy bias, this group of already-insured people may not even understand the specifics of reimbursement for health care services, and are more likely to incur high expenditures after misuse of health care services, thus pushing up the risk of CHE.

Furthermore, regional economic development regulates the intensity of the relationship between DPE and CHE through the dual pathways of income effects and health-system effectiveness. Relevant cross-country comparison points out that economically backward areas are more prone to CHE because of poor access to healthcare services, weak household payment capacity, and the absence of prepaid insurance (40). Empirical data from central and western China confirm this pattern; although the incidence of CHE in these regions is comparable to that in the eastern regions (13.4% vs. 13.3%), households in central and western China experience lower hospitalization rates but higher costs. This disparity reflects a shortage of medical resources, compelling patients to seek treatment across regions and indirectly increasing out-of-pocket expenses costs (41). This finding suggests that the impact of economic development on CHE is non-linear. In poor areas, income constraints are the dominant factor, whereas in affluent areas, distortions in the health service market may offset economic advantages. Relevant studies have shown that health literacy is significantly lower in individuals with low levels of education compared to individuals with high levels of education (42), and are more likely to lead to poorer health conditions (43), Relatively higher exposure to health risks (44). In reality, individuals in rural areas and economically disadvantaged areas have lower education levels than those in urban areas and economically developed areas, and their poor health literacy leads to their vulnerability to health insurance policies, and any action taken by the policy implementation layer will have a significant impact on them, which explains why they are more susceptible to DPE impacts. Differences in DPE across different regions further amplify the economic gradient effect. DPE emerged as a key variable and may further deepen the aforementioned impacts. Based on this, a second-stage hypothesis was proposed.

*H2a*: DPE affects residents in rural areas and increases their risk of developing CHE.

*H2b*: People covered by health insurance are affected by DPE and are at a higher risk of developing CHE.

*H2c*: Compared with economically developed regions, DPE in underdeveloped and remote areas has a stronger correlation with CHE.

From the perspective of behavioral decision-making, DPE tends to create a typical information asymmetry dilemma (45), causing residents to receive fragmented policy information during the enrollment process. This results in systematic cognitive biases regarding core issues such as reimbursement rates for first-level consultations at primary healthcare facilities and referral processes, exacerbating the cognitive gaps of insured residents concerning key policies like DRG payment standards and deductible adjustment rules. In the face of uncertain reimbursement expectations, coupled with a persistent belief that the quality of services at primary healthcare facilities falls below psychological expectations, residents adopt a defensive healthcare-seeking strategy of treating minor illnesses as major ones, preferring to select tertiary hospitals that offer a greater margin of medical safety, even if this incurs additional transport costs and opportunity costs in time, thus creating a significant ratchet effect (46). Regarding direct cost structures, tertiary hospitals, in contrast to primary healthcare facilities, concentrate more on medical resources, resulting in significantly higher average outpatient and inpatient costs (47, 48). Data from China’s Health Security Statistical Yearbook also support this point (taking the latest 2024 data as an example, the average inpatient costs at tertiary, secondary, and primary healthcare facilities were 12,765yuan, 6,205 yuan, and 2,943 yuan, respectively). The diagnosis and treatment of numerous minor, common, and frequently occurring illnesses also tend to lead to an imbalance in medical resource allocation (49), triggering a siphoning effect caused by concentrated consultations. Consequently, although residents have health insurance protection, they still face higher out-of-pocket expenditures during diagnosis and treatment. At this point, the preference to seek treatment at large hospitals can easily cause household medical expenditures to exceed affordable limits (50), inducing the risk of CHE. The preference for a healthcare institution (PHI) is an intermediary in the logical relationship between DPE and CHE. Based on this, we propose the following third-stage hypothesis:

*H3*: PHI plays an intermediary role between DPE and CHE.

**Figure 1:**
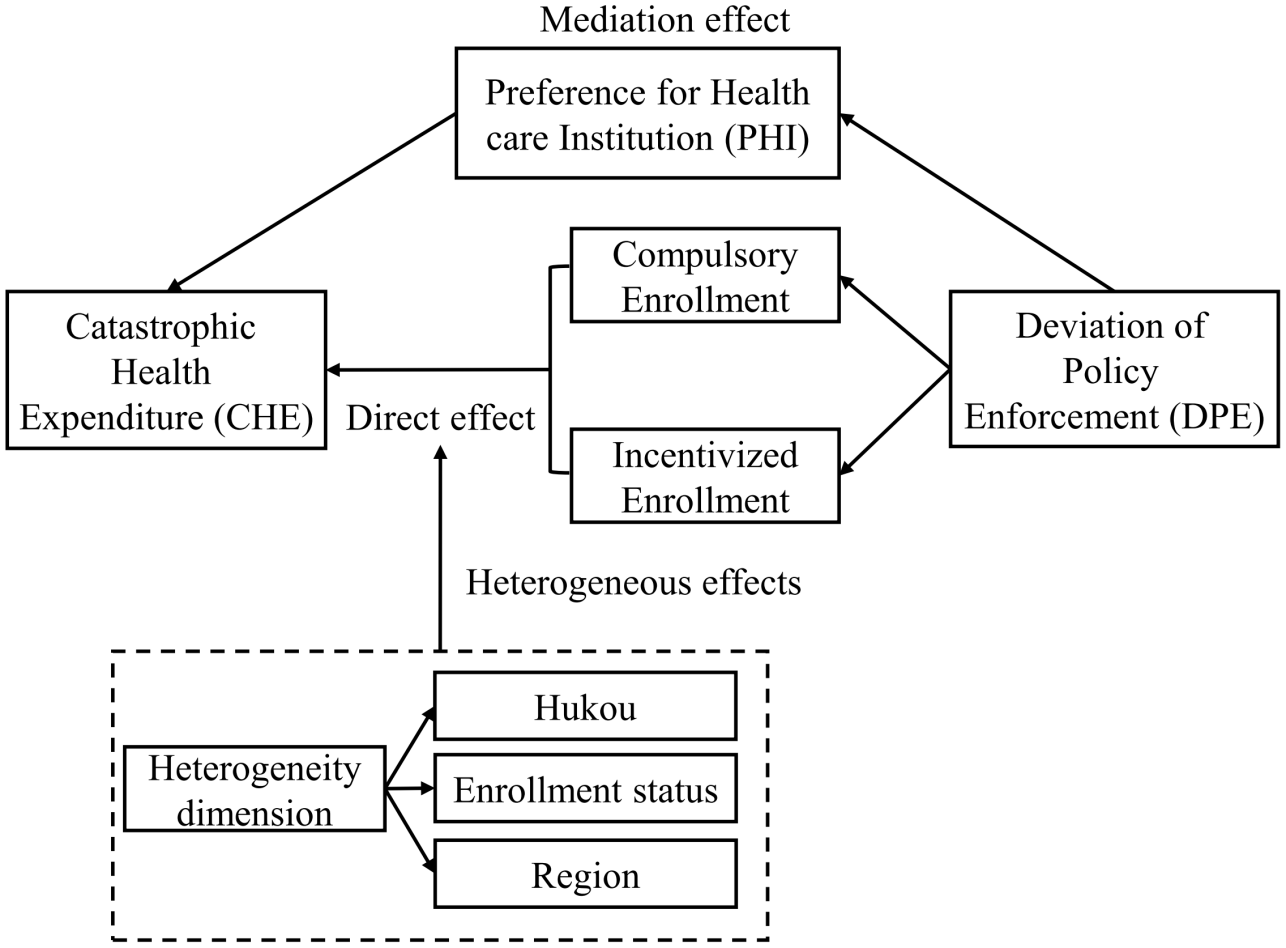
Research and analysis framework.

The analytical framework for our research is shown in Figure 1.

## 3 Methodology

### 3.1 Data and variable definitions

#### 3.1.1 Data description

The data used in our study originated from a social survey conducted between September and December 2024, focusing on the implementation and participation in urban and rural resident medical insurance policies in Sichuan Province, China. To maximize the representation of geographical differences and the disparity between developed cities and less-developed ethnic areas, thereby enhancing the national referential value of the study regarding the effectiveness of medical insurance policy implementation, the survey sampled five cities and prefectures—Chengdu, Zigong, Nanchong, Aba Tibetan and Qiang Autonomous Prefecture, and Liangshan Yi Autonomous Prefecture—as representatives. These locations were selected based on the geographical divisions of Sichuan Province and the economic development gradients of various cities and prefectures. The target respondents were residents of urban and rural areas who had not participated in employee health insurance. The sample was fully representative and typical. The survey collected 1,555 samples. After removing missing values, invalid questionnaires and consolidating relevant indicator items, a final sample size of 1,460 was obtained. We conducted reliability tests on the primary indicators of the research data, with results showing a Cronbach’s alpha of 0.8228, which exceeds 0.8. Bartlett’s sphere test yielded a *p*-value significantly less than 0.01, indicating its suitability for factor analysis with good reliability validity. All samples were anonymized, and no personal information was provided regarding their privacy. All the residents who participated in the survey were informed of the research terms and related content.

#### 3.1.2 Variable explanation

##### 3.1.2.1 Independent variables

DPE in resident health insurance refers to behaviors observed during the grassroots implementation of the resident health insurance policy, such as publicity, notifying residents about insurance enrollment, and premium payments, that diverge from the principles or regulations of the policy. The specific manifestations are primarily reflected through two indicators: the extent of influence of the village community’s mandatory requirement for insurance enrollment (DPE-1) and the extent of influence of the community’s related incentive measures after enrollment (DPE-2). The original questions were “To what extent did the village community’s behavior of forcing you to pay health insurance premiums affect your final decision to enroll?” and “To what extent did the village community’s behavior of inducing you to pay health insurance premiums affect your final decision to enroll?” Both indicator variables are assigned values ranging from 1 to 5, with lower values indicating a greater influence. Assigning weights to each indicator and converting multi-objective problems into a single scalar optimization are common decision-making methods in practical scenarios. Standardized weights also significantly impact the results, reducing the disparity between indicators and minimizing outcome deviation (51–53). We combined DPE-1 and DPE-2 with an equal weighting of 0.5 to form the DPE variable. As both sub-variables show a consistent directional change, the final composite explanatory variable is also assigned values from 1 to 5, with lower values representing a stronger influence.

##### 3.1.2.2 Dependent variable

CHE. According to the World Health Organization (WHO), CHE risk is identified when a family’s mandatory health expenditure (HE) is greater than or equal to 40% of total family expenditure (TE) after deducting basic living expenses, specifically food expenditure (FE). In this survey, total family expenditure, family food expenditure, and family mandatory health expenditure were all calculated annually, with family mandatory health expenditure not including costs after insurance reimbursement. Based on this, the calculation formula and meaning of the assignment for CHE in this study are indicated in Equation 1.


(1) $$\mathrm{CHE}=\mathrm{HE}/(\mathrm{TE}-\mathrm{FE})*100\%\{\begin{array}{l} 0,\mathrm{CHE}<40\%\\ 1,\mathrm{CHE}\geq 40\% \end{array}$$

If the calculated result of the previously mentioned variables is below 0.4, the CHE value is set to 0, indicating that a household does not face CHE. In contrast, if the result is 0.4 or higher, the CHE value is 1, showing that the household has encountered CHE.

##### 3.1.2.3 Control variables

Basic variables such as sex, age, hukou status, educational level, and annual household income are frequently included in CHE studies (38, 54, 55). The common practice of applying a logarithmic transformation to income to mitigate the interference of extreme values was also noted (56). The primary variables and models used in our study are non-linear. Since the related household expenditure variables are included as comprehensive factors of the dependent variable, we decided to retain the original income variable to preserve consistency in the explanatory results. Given that the survey area covers ethnic minority regions, the ethnicity variable may also impact the outcomes and should be included among the control variables. Furthermore, reflecting real-world conditions and the Chinese context, the number of family members often positively correlates with household expenditure. Special groups, such as individuals in extreme poverty, low-income households, and those under poverty prevention monitoring, are more likely to experience CHE due to low income, which may affect the ideological awareness of the sample individuals, resulting in differing perceptions of grassroots policies and their enforcement. Considering these factors, it is essential to control the aforementioned variables.

Additionally, our study identifies “health insurance satisfactory perception” as the instrumental variable. The original question asked was, “How satisfied are you with the current resident health insurance system?” with ratings ranging from 1 to 5, where a higher value indicates a lower level of satisfaction. The mediating variable is “preference for healthcare institution (PHI).” The original question posed was, “When you or your family members become ill, what type of healthcare institution do you typically choose for medical treatment?” with ratings from 1 to 9, in which a higher value signifies a higher level of the healthcare institution.

The measurement coding for each variable indicator and the descriptive statistical results are presented in Table 1.

**Table 1:** Descriptive statistics of main variables (*N* = 1,460).

Variable indicators	Measure coded	Percentage(n)/Mean	Standard Deviation	Min	Max
CHE^a^	0 = No, 1 = Yes	63.49% (927), 36.51% (533)	0.4816	/	/
DPE-1^b^	1 = significant impact, 2 = greater impact, 3 = general impact, 4 = less impact, 5 = almost no impact	2.81% (41), 16.03% (234), 7.53% (110), 28.42% (415), 45.21% (660)	1.1922	/	/
DPE-2^c^	1 = significant impact, 2 = greater impact, 3 = general impact, 4 = less impact, 5 = almost no impact	2.88% (42), 17.53% (256), 7.95% (116), 22.19% (324), 49.45% (722)	1.2389	/	/
Gender	1 = Male, 2 = Female	40.89% (597), 59.11% (863)	0.4918	/	/
Age	Record value	56.1158	15.9952	14	105
Hukou	Official residency documentation 1 = Rural hukou, 2 = Urban hukou, 3 = No hukou, 4 = Other types	85.14% (1,243), 13.97% (204), 0.68% (10), 0.21% (3)	0.4003	/	/
Ethnic group	1 = Han ethnicity, 2–9 = Other ethnicities	74.73% (1,091), 25.27% (369)	1.3187	/	/
Education	1–2 = Low education level, 3–4 = Medium education level, 5–6 = High education level	52.81% (771), 39.52% (577), 7.67% (112)	1.2291	/	/
Political affiliation	1 = masses, 2 = League member, 3 = Communist	90.55% (1,322), 3.90% (57), 5.55% (81)	0.4885	/	/
Number of family members	Record value	4.1514	1.9319	1	13
Annual household income	Record value	52946.09	51387.47	0	600,000
Special group	0 = No, 1 = Yes	93.15% (1,360), 6.85% (100)	0.2527	/	/
Health insurance satisfactory perception	1 = Very high, 2 = Relatively high, 3 = Moderate, 4 = Relatively low, 5 = Very low	9.73% (142), 55.62% (812), 11.16% (163), 19.86% (290), 3.63% (53)	1.0295	/	/
PHI^d^	1 = Pharmacy, 2 = Private clinics, 3 = Community health rooms, 4 = Township health centers, 5 = County hospital, 6 = City hospital, 7 = Specialized hospital, 8 = Provincial hospital 9 = Other higher-level hospitals	25.96% (379),16.71% (244), 3.29% (48), 18.29% (267), 21.85% (319), 11.71% (171), 1.51% (22), 0.27% (4), 0.41% (6)	1.9051	/	/

^a^ Catastrophic health expenditure, ^b^ Compulsory enrollment, ^c^ Incentivized enrollment, ^d^ Preference for healthcare institutions.

### 3.2 Analysis model

Regarding specific characteristics, CHE is classified into 0 and 1, indicating the occurrence or non-occurrence of CHE, a binary variable. Furthermore, after testing, CHE exhibited non-linear characteristics to DPE-1 and DPE-2. Consequently, a binary probit model is appropriate for conducting a regression analysis to explain the parameters to be estimated. The following formula gives the specific logic of an action:


(2) $${\mathrm{CHE}}_{i}=\beta_{0}+\beta_{1}\mathrm{DPE}-1_{i}+\beta_{2}{\text{ΣCtrls}}_{i}+e_{i}$$

(3) $${\mathrm{CHE}}_{i}=\lambda_{0}+\lambda_{1}\mathrm{DPE}-2_{i}+\lambda_{2}{\text{ΣCtrls}}_{i}+e_{i}$$

Equations 2, 3 represent the regression estimation models for *DPE-1* and *DPE-2* to *CHE*, respectively. In these equations, *CHE_i_* denotes the occurrence of CHE for the individual sample; the explanatory variables *DPE-1* and *DPE-2* represent the “degree of influence of the village community’s mandatory requirement to participate in insurance” and the “degree of influence of the village community’s related incentive measures after enrollment in insurance,” respectively. *ΣCtrls_i_* denotes the sum of the control variables, including individual characteristics and household income, etc. *e_i_*, and *ν_i_* and *η_i_* in Equations 6, 7 represent other disturbance terms. The coefficients *β*, *λ*, *γ* in Equations 4, 7, and *α* in Equation 6 are the regression coefficients of the model.


(4) $${\mathrm{CHE}}_{i}=\gamma_{0}+\gamma_{1}{\mathrm{DPE}}_{i}+\gamma_{2}{\text{ΣCtrls}}_{i}+e_{i}$$

(5) $${\mathrm{DPE}}_{i}=0.5^{*}\mathrm{DPE}-1_{i}+0.5^{*}\mathrm{DPE}-2_{i}$$

Equation 4 represents the regression estimation model for the relationship between *DPE* and *CHE*. The sum of the half-weighted values of *DPE-1* and *DPE-2* constitutes *DPE*, as indicated by Equation 5, with all other variables remaining consistent.


(6) $${\mathrm{PHI}}_{i}=\alpha_{0}+\alpha_{1}{\mathrm{DPE}}_{i}+\alpha_{2}\;{\text{ΣCtrls}}_{i}+\nu_{i}$$

(7) $${\mathrm{CHE}}_{i}=\gamma_{0}+\gamma_{1}{\mathrm{DPE}}_{i}+\gamma_{2}\;{\mathrm{PHI}}_{i}+\gamma_{3}{\text{ΣCtrls}}_{i}+\eta_{i}$$

Equation 6 represents the regression model for the mediating variable *PHI* and Equation 7 represents the mechanism model of the mediating effect of *PHI* between *DPE* and *CHE*. In these models, *PHI_i_* denotes residents’ healthcare institution preferences.

## 4 Results

### 4.1 Benchmark model regression

A binary probit model was used to conduct benchmark regression analysis. After incorporating individual variables and some family variables as control factors, the results of Model 1 in Table 2 indicate that the enforcement method of the health insurance policy, in which village committees or communities mandate resident participation, has a significant impact on household CHE (*β* = −0.0658, *p* < 0.05). Model 2 results show that the health insurance policy enforcement method, which includes related incentives after resident enrollment, also significantly affects household CHE (*λ* = −0.0597, *p* < 0.05). Model 3 weights the two policy enforcement methods and synthesizes the core explanatory variable “DPE.” The results suggest that the deviation in enforcing urban and rural resident medical insurance policies is negatively significant at the 5% level to the observed sample household CHE (*γ* = −0.0745, *p* < 0.05). This means that for every one-unit increase in the degree of policy deviation, the probability of a household experiencing CHE rises significantly by 7.45%. From the perspective of the benchmark regression, the results of the first and second types of deviation factors provide preliminary evidence for our research. Specifically, when residents are required to enroll in health insurance, it may place an additional economic burden on families with limited financial resources. Suppose a family cannot afford insurance premiums but complies with regulations. In that case, it may limit the consumption of other essential goods and possibly resort to borrowing or other means of paying premiums, thereby increasing the family’s economic pressure. From the perspective of adverse selection in health insurance, mandatory enrollment may result in a higher tendency for residents with poorer health to enroll, while those in better health may choose not to enroll or find other ways to avoid it (57, 58). The incentive-based policy enforcement method may exacerbate inequality among the population, leading to some residents who are willing to participate not fully understanding the true intent and coverage of the policy. This decreases their trust in the policy and affects their willingness to participate. This could result in higher out-of-pocket payment proportions or stricter compensation conditions when insured residents require medical services, thereby increasing the risk of CHE. This result validates H1a, H1b, and H1c.

**Table 2:** Benchmark model regression results.

Main variables	Model 1	Model 2	Model 3
	CHE^a^	CHE	CHE
DPE-1^b^	−0.0658^**^ (0.0289)		
DPE-2^c^		−0.0597^**^ (0.0279)	
DPE^d^			−0.0745^**^ (0.0309)
Gender	0.0448 (0.0703)	0.0393 (0.0703)	0.0418 (0.0703)
Age	0.0059^**^ (0.0027)	0.0056^**^ (0.0027)	0.0057^**^ (0.0027)
Hukou	−0.133 (0.0898)	−0.129 (0.0899)	−0.131 (0.0899)
Ethnic group	0.0057 (0.0287)	0.0052 (0.0286)	0.0046 (0.0287)
Education	−0.0700^*^ (0.0361)	−0.0769^**^ (0.0365)	−0.0754^**^ (0.0363)
Political affiliation	0.199^***^ (0.0757)	0.202^***^ (0.0757)	0.202^***^ (0.0757)
Number of family members	0.0083 (0.0188)	0.0088 (0.0189)	0.0084 (0.0189)
Annual household income	−0.0000^***^ (0.0000)	−0.0000^***^ (0.0000)	−0.0000^***^ (0.0000)
Special group	0.189 (0.136)	0.209 (0.135)	0.197 (0.136)
*N*	1,460	1,460	1,460
LR chi^2^	77.38	76.78	77.99
Pseudo *R*^2^/*R*^2^	0.040	0.040	0.041

^a^catastrophic health expenditure; ^b^compulsory enrollment; ^c^incentivized enrollment; ^d^weighted index (0.5 × DPE-1 + 0.5 × DPE-2).

Standard errors in parentheses.

^*^ *p* < 0.1, ^**^ *p* < 0.05, ^***^ *p* < 0.01.

### 4.2 Endogeneity test

Although the benchmark regression analysis controls for factors that may influence the final outcomes, other unobservable factors could interfere with the research results, and an endogeneity bias may still exist. Using instrumental variables (IV) has proven to be an effective method (59). Health insurance enrollment, as a policy concerning people’s livelihoods, involves the enforcement and feedback of public policies. In the policy enforcement phase, evidence from this survey suggests that grassroots implementers, owing to higher-level policy pressure, may enforce mandatory enrollment in health insurance or establish paid mechanisms. The direct consequence is increased distrust of the policy among residents (60, 61), leading to decreased satisfaction with the health insurance policy. Moreover, CHE is closely linked to population, age, region, individual health status, and income. While medical reforms impact CHE for low-income families to some degree, no clear evidence indicates a relationship between CHE and residents’ satisfaction with health insurance. Satisfaction among residents concerning health policies arises from assessing the fairness between their expectations of the system and their actual experiences (62). CHE may impact the long-term financial situation of a family, but this does not necessarily immediately reflect residents’ satisfaction with health insurance, which may be influenced more by short-term experiences. In some cases, factors such as social support (63) and family background (64) may buffer the impact of CHE, resulting in no direct relationship.

Although the validity of the selected instrumental variable “medical insurance satisfaction” can be theoretically explained, further verification is still required. We employed the Two-Stage Least Squares (2SLS) method for the first-stage regression and the Ordinary Least Squares (OLS) method for the second-stage test. The first-stage regression results of 2SLS for Models 1–3 in Table 3 indicate that the *p*-values corresponding to the test coefficient values are not significant at the 1% level when analyzing the instrumental variable to be estimated and the endogenous explanatory variable, and the relationship between the instrumental variable and the observed endogenous variable has not been fully clarified (*β* = 0.887, *p* > 0.1; *λ* = 1.717, *p* > 0.1; *γ* = 1.170, *p* > 0.1). In the second-stage test, OLS regression was used again to conduct the Hausman test for verification. The results of Models 4–6 show that the IV regression coefficients exhibit strong significance (*β* = −0.023, *p* < 0.05; *λ* = −0.021, *p* < 0.05; *γ* = −0.026, *p* < 0.05). The Hausman test results suggest that we cannot reject the null hypothesis of systematic differences between OLS and 2SLS estimates at the statistical level (the corresponding *p*-values are 0.059, 0.063, and 0.060, all greater than 0.05). After testing with the residuals from the first-stage regression, the report shows that the p-values of the coefficients of the remaining explanatory variables are all greater than 0.05, indicating that the explanatory variables in the model have almost no explanatory power for the residuals. This demonstrates that the instrumental variable is not correlated with the error term of the model, and its exogeneity is verified, supporting the validity of the instrumental variable.

**Table 3:** Endogenous test results.

Variables	Model 1 (2SLS ^a^)	Model 2 (2SLS)	Model 3 (2SLS)	Model 4 (OLS ^b^)	Model 5 (OLS)	Model 6 (OLS)
	2SLS Phase one regression			2SLS Two-stage test		
	CHE^c^	CHE	CHE	CHE	CHE	CHE
DPE-1^d^	0.887 (1.200)			−0.023^**^ (0.0105)		
DPE-2^e^		1.717 (4.300)			−0.021^**^ (0.0101)	
DPE^f^			1.170 (1.888)			−0.026^**^ (0.0112)
Constant term	−3.459 (5.092)	−8.052 (20.93)	−5.024 (8.598)	0.397^***^ (0.120)	0.405^***^ (0.122)	0.419^***^ (0.123)
Wald chi^2^	11.51	3.36	8.09	11.51	3.36	8.09
*F*-statistic				7.23	7.21	7.30
Hausman test				3.55	3.46	3.53
Root MSE	1.172	2.170	1.398	0.472	0.472	0.472
Controls	YES	YES	YES	YES	YES	YES
*R* ^2^				0.048	0.047	0.048
*N*	1,460	1,460	1,460	1,460	1,460	1,460

^a^ two-stage least squares, ^b^ ordinary least squares, ^c^ catastrophic health expenditure; ^d^ compulsory enrollment; ^e^ incentivized enrollment; ^f^ weighted index (0.5 × DPE-1 + 0.5 × DPE-2).

### 4.3 Robustness tests

#### 4.3.1 Changing the benchmark regression model

In econometric research, Probit and Logit models are similar in their applicability (65, 66). In the first stage, we use a logit model for robustness testing. The results are shown in Table 4, in Models 1–3. At the 5% significance level, DPE-1, DPE-2, DPE, and CHE are significant, confirming the robustness of the benchmark model. In the second stage, we continue testing by modifying the explanatory variables.

**Table 4:** Robustness test results.

Main variables	Model 1 (logit)	Model 2 (logit)	Model 3 (logit)	Model 4 (probit)	Model 5 (probit)	Model 6 (probit)	Model 7 (probit)	Model 8 (probit)
	CHE^a^	CHE	CHE	CHE	CHE	CHE	CHE	CHE
DPE-1^b^	−0.104^**^ (0.0472)					−0.0952^**^ (0.0372)		
DPE-2^c^		−0.0982^**^ (0.0455)					−0.0758^**^ (0.0373)	
DPE^d^			−0.120^**^ (0.0505)					−0.102^**^ (0.0408)
DPE-3^e^				−0.0532^*^ (0.0285)				
DPE-4^f^					−0.0763^**^ (0.0322)			
Controls	YES	YES	YES	YES	YES	YES	YES	YES
N	1,460	1,460	1,460	1,460	1,460	856	856	856
LR chi^2^	77.62	77.40	78.40	75.67	77.82	30.64	28.22	30.40
Pseudo *R*^2^	0.041	0.040	0.041	0.040	0.041	0.030	0.028	0.030

^a^ catastrophic health expenditure, ^b^ compulsory enrollment, ^c^ incentivized enrollment, ^d^ weighted index (0.5 × DPE-1 + 0.5 × DPE-2), ^e^ alienation of health insurance policy propaganda, ^f^ weighted index (1/3 × DPE-1 + 1/3 × DPE-2 + 1/3 × DPE-3).

Standard errors in parentheses.

^*^ *p* < 0.1, ^**^ *p* < 0.05, ^***^ *p* < 0.01.

#### 4.3.2 Changing the weights of explanatory variables

In practice, promoting policy often overlaps with executing it. This overlap can lead to misunderstandings of policies, selective promotion, and mere repetition, which may result in inconsistencies in execution. Consequently, during the second stage of the test, we incorporate the extent of deviation in health insurance policy promotion as an explanatory variable. The specific assignment mirrors the approach used in the previous two policy deviation methods and serves as a sub-variable for the DPE, with equally adjusted weight proportions of 1/3. The findings in Table 4, Model 5, show that the extent of policy promotion deviation is significant at the 10% level, while the newly established DPE variable achieves significance at the 5% level with CHE, confirming the explanatory robustness variables.

#### 4.3.3 Changing the sample

Our survey also statistically accounted for the physical health conditions of residents in the indicator design. Based on the research needs, we excluded individuals with good health and retained those with moderate to poor health to form a new sample for robust regression. The results, as shown in Table 4, Models 6–8, indicate that all DPE indicators and CHE exhibit strong significance (*β* = −0.0952, *p* < 0.05; *λ* = −0.0758, *p* < 0.05; *γ* = −0.102, *p* < 0.05). This suggests that the risk of CHE due to DPE is also present in patients with poor health. This may be because individuals in poor health are more likely to face high medical expenses, and the deviation of health insurance policies enforcement may result in a lack of policy understanding for the unhealthy residents. This can lead to an inability to utilize health insurance resources fully or to make decisions that are unfavorable to themselves, which in turn increases the risk of CHE.

### 4.4 Heterogeneity tests

#### 4.4.1 Hukou dimension

Due to the influence of regional administrative jurisdiction, the hukou status of the samples observed in our study was categorized as rural, urban, or no hukou. After excluding samples without a hukou, we classified individuals into rural and urban groups for heterogeneity testing. Table 5, Models 1–2, shows that the regression coefficient of DPE on CHE for rural individuals is significant at the 5% level, while it is not significant for urban individuals. This indicates that rural residents are at a higher risk of experiencing CHE due to the influence of DPE compared to urban residents. In the Chinese context, village officials serve as policy implementers in rural China, and their dual roles (67) may create a risk of distortion in enforcing public policies at the grassroots level, resulting in DPE. The income level of rural residents is generally lower, and their economic situation is relatively challenging. Rural residents often struggle to cope with high health expenses. Although health security systems such as the New Rural Cooperative Medical Scheme (NRCMS) have alleviated the health burden on rural residents to some extent (68), the NRCMS compensation policy has not significantly improved the health expense burden for middle-aged and older rural populations. The setting of deductibles and limitations on compensation ratios and limits affect the effectiveness of health security for rural residents (69), leading to a higher likelihood of CHE when facing policy execution deviations. This validates H2a.

**Table 5:** Heterogeneity tests results of hukou status and insurance enrollment status.

Main variables	Model 1 (Rural Hukou)	Model 2 (Urban Hukou)	Model 3 (Enrollment)	Model 4 (Unenrollment)
	CHE^a^	CHE	CHE	CHE
DPE^b^	−0.0821^**^ (0.0331)	−0.0572 (0.0947)	−0.0702^**^ (0.0315)	−0.266 (0.197)
Controls	YES	YES	YES	YES
*N*	1,243	204	1,400	60
LR chi^2^	56.10	18.85	78.73	8.76
Pseudo *R*^2^	0.034	0.078	0.043	0.122

^a^ catastrophic health expenditure, ^b^ weighted index (0.5 × DPE-1 + 0.5 × DPE-2).

Standard errors in parentheses.

* *p* < 0.1, ** *p* < 0.05, *** *p* < 0.01.

#### 4.4.2 Insurance enrollment dimension

Related research indicates that individuals who are not enrolled in health insurance have a weaker ability to withstand risks when facing diseases than those who are insured and are more likely to experience CHE due to the high health costs incurred from diagnosis and treatment (70). The heterogeneity comparison results shown in Table 5, Models 3–4 indicate that among those with health insurance, the impact of DPE on CHE is significantly evident at the 5% level. Conversely, DPE shows no significant effect on CHE for those without health insurance, which contradicts earlier research findings. One possible explanation is that the assurance provided by health insurance may lead the enrolled group to underestimate risks, causing them to overlook the implications of changes in disease prevalence and insurance policies. This lack of awareness can exacerbate challenges for this group, as despite being insured, they may not fully comprehend their insurance coverage limits, resulting in out-of-pocket healthcare expenses and a significant financial burden on families, ultimately leading to CHE. This validates H2b.

#### 4.4.3 Region dimension

Our research data were obtained from five cities and prefectures in Sichuan Province: Chengdu, Zigong, Nanchong, Liangshan Yi Autonomous Prefecture, Aba Tibet, and Qiang Autonomous Prefecture. As shown in Table 6, Models 4–5, from the perspective of regional heterogeneity, the regression analysis of DPE on CHE in the two minority autonomous prefectures of Liangshan and Aba shows strong significance (*γ* = −0.227, *p* < 0.01; *γ* = −0.183, *p* < 0.05), whereas Chengdu, Zigong, and Nanchong do not exhibit significant correlation. Considering the current conditions in the cities and prefectures of Sichuan Province, the possible reasons are as follows. On the one hand, Aba and Liangshan are situated in Sichuan’s western and southwestern regions, with complex terrain and harsh climates, increasing the difficulty and cost of medical services. Moreover, due to their relatively low levels of economic development, residents’ income levels are also quite low, making it easier for them to fall into the predicament of CHE when faced with high medical costs. Chengdu, Zigong, and Nanchong, located in the central and eastern parts of the Sichuan Basin, have higher levels of economic development, and the enforcement of health insurance policies there may be smoother, with residents better able to bear health expenses. In contrast, Aba and Liangshan are areas where ethnic minorities reside, including many Tibetans, Yi, and other ethnic groups. These populations’ traditional lifestyles, cultural customs, and religious beliefs may differ from those of mainstream society, influencing the acceptance and effectiveness of health insurance policies. Due to language and cultural factors, the promotion of health insurance in these areas may be insufficient, and local officials may be more likely to deviate from policy enforcement, resulting in a lack of understanding of these policies among the local ethnic minority community residents. They may face difficulties comprehending and utilizing health insurance policies, thereby being unable to utilize insurance to alleviate the burden of health expenses fully. In Chengdu, Zigong, and Nanchong, where the Han population is predominant, cultural customs and lifestyles are relatively uniform, making the dissemination and enforcement of health insurance policies smoother and easier for residents to understand and utilize in reducing the burden of health expenses, thus validating H2c.

**Table 6:** Heterogeneity tests results of regions.

Main variables	Model 1 (Chengdu)	Model 2 (Zigong)	Model 3 (Nanchong)	Model 4 (Liangshan)	Model 5 (Aba)
	CHE^a^	CHE	CHE	CHE	CHE
DPE^b^	−0.0033 (0.0672)	−0.0274 (0.0720)	0.106 (0.0809)	−0.227^***^ (0.0659)	−0.183^**^ (0.0885)
Controls	YES	YES	YES	YES	YES
*N*	309	264	314	324	248
LR chi^2^	48.31	29.34	44.62	21.31	7.65
Pseudo *R*^2^	0.019	0.060	0.106	0.120	0.092

^a^ catastrophic health expenditure, ^b^ weighted index (0.5 × DPE-1 + 0.5 × DPE-2).

Standard errors in parentheses.

^*^ *p* < 0.1, ^**^ *p* < 0.05, ^***^ *p* < 0.01.

### 4.5 Mediation effect test

Our survey inquired about residents’ PHI and used it as a mediating variable. As shown in Table 7, Models 1–3 were analyzed using a three-step method for the mediation effect regression. The results of Step 1 indicate that the impact of DPE on CHE is significantly negative at the 5% level. The results of Step 2 show that DPE has a significant negative effect on PHI at the 1% level. Step 3, which includes CHE and PHI in the regression analysis, reveals that the regression coefficient is significantly negative at the 10% level. The significance across all three steps is consistently negative, similar to the baseline regression, indicating that PHI mediates between DPE and CHE. Thus, DPE indirectly leads to an increase in CHE by influencing PHI, validating H3.

**Table 7:** Mediation effect tests results of PHI.

Main variables	Model 1 (Step 1)	Model 2 (Step 2)	Model 3 (Step 3)
	CHE^a^	PHI^c^	CHE
DPE^b^	−0.0745^**^ (0.0309)	−0.0813^***^ (0.0249)	−0.0587^*^ (0.0313)
PHI			0.110^***^ (0.0186)
Controls	YES	YES	YES
*N*	1,460	1,460	1,460
LR chi^2^	77.99	52.38	113.20
Pseudo *R*^2^	0.041	0.010	0.059

^a^ Catastrophic health expenditure, ^b^ Weighted index (0.5 × DPE-1 + 0.5 × DPE-2), ^c^ Preferences for healthcare institutions.

Standard errors in parentheses.

* *p* < 0.1, ** *p* < 0.05, *** *p* < 0.01.

## 5 Discussion

Based on survey data from five cities in Sichuan Province, this study reveals a significant association between DPE and CHE: DPE significantly increases the risk of CHE, with coercive insurance participation and incentivized measures raising the likelihood of CHE in households by 7.45%. Regional and group heterogeneity is evident, with higher CHE risks in rural areas and regions populated by ethnic minorities, reflecting the combined effects of economic vulnerability and cultural differences. Additionally, PHI mediates the impact of DPE on CHE, indicating that DPE indirectly heightens the medical burden through its influence on residents’ healthcare-seeking behaviors.

To address these findings, the following policy optimizations are recommended.

First, grassroots health insurance policies should be standardized in their enforcement, and supervisory and accountability mechanisms must be strengthened. A negative list system should be established to minimize the impact of policy deviations caused by a pressure-based system that explicitly prohibits coercive insurance participation and incentivized measures. Consider incorporating policy enforcement standards into the evaluations of grassroots officials, introducing third-party assessments, and enhancing transparency by having independent institutions regularly conduct audit policies. Establish a feedback loop by utilizing the Singapore Health Promotion Board model (71), which involves setting up grassroots health insurance consultation service points to collect resident feedback and optimize policy enforcement in real-time.

Second, the rapid increase in health insurance premium standards may escalate household financial pressures and potentially trigger CHE crises. Therefore, it is essential to consider incorporating differentiated premiums into the health insurance system design and dynamically adjusting contribution rates based on family income to avoid a one-size-fits-all approach that burdens the poor families. Prior to implementing the premium differentiation policy for low-income groups, a detailed financial cost–benefit analysis is required, including assessments of premium subsidy scale, health insurance fund balance, and long-term fiscal pressure. A cap on premium subsidies should be established to avoid excessive reliance on fiscal appropriations. Meanwhile, diversified funding channels such as government special subsidies, social charitable donations, and investment income from medical insurance funds should be explored to support the long-term implementation of the premium differentiation policy and ensure financial sustainability.

Third, the central and local governments should increase special transfer payments to ethnic minority and rural areas to ensure adequate protection for vulnerable groups and reduce their out-of-pocket burden. Meanwhile, differentiated policy implementation strategies should be formulated for different groups. Specifically, for urban residents, it is necessary to optimize the medical insurance publicity and service network, utilize digital means to enhance policy transparency, and reduce information asymmetry. For rural residents, efforts should be made to strengthen the construction of grassroots medical facilities, improve the quality of medical services, and promote a medical insurance financing mechanism suitable for rural characteristics, such as the “fixed subsidy + individual payment” model, to alleviate the burden on farmers. For residents in ethnic minority areas, it is essential to respect ethnic cultural customs, strengthen multilingual publicity of medical insurance policies, and produce brochures and videos in ethnic languages to ensure policy comprehension and acceptance.

In addition, payment reform should be deepened and risk sharing should be strengthened. Promote graded diagnosis, treatment, and DRG reform while advocating for decentralized healthcare to reduce defensive health practices and moral hazards. Further optimizing the health insurance directory and ceiling design will increase reimbursement rates for chronic diseases and major illnesses. Concurrently, we should vigorously develop supplementary health insurance, encourage the integration of commercial insurance with basic health insurance, and diversify CHE risks through a dual approach of government and market involvement.

## 6 Conclusion

Our survey only covered five cities in Sichuan Province, so caution should be taken when generalizing the conclusions nationally, especially since eastern developed regions may exhibit different patterns. However, due to the time cross-section limitation, our cross-sectional data may not fully capture the long-term dynamic relationship between DPE and CHE, such as the lag effect following the policy adjustments. Families’ ability to withstand risk may moderate the relationship between DPE and CHE. Due to limitations in data availability, our study has not yet explored this dimension. In addition, due to the limitations of the availability of our research data, the sample size of groups in different regions and social realities has disparities, which may bias the discussion on specific categorization.

Future research should consider broadening the study’s scope to include data from additional provinces, and balancing the data sample sizes of urban and rural areas as well as different individual characteristics to enhance the accuracy of the study, so as to analyze more deeply the regional collaborative governance strategies in the context of urban–rural integration and rural revitalization. In addition, consider using tracking survey designs to uncover the causal relationship between policy changes and CHE, such as the effects of healthcare reforms, commercial insurance, and social support networks on household economic resilience; and incorporating behavioral economics experiments to quantify psychological biases in residents’ insurance enrollment decisions, providing micro-level evidence for policy design.

## Data Availability

The original contributions presented in the study are included in the article/supplementary material, further inquiries can be directed to the corresponding author.

## Footnotes

^1^Content evidence was collated through field interviews with residents of five cities in Sichuan Province.
